# Percutaneous nephrolithotomy for renal stones combined with laser endoscopy for ipsilateral renal cysts: a case report and literature review

**DOI:** 10.3389/fsurg.2025.1599309

**Published:** 2025-05-16

**Authors:** Shuxin Li, Fulin Wang, Hongliang Cao, Yueqiu Zhang, Binbin Wang, Gengchen Huang, Wei Wei

**Affiliations:** ^1^ Department of Urology, The First Hospital of Jilin University, Changchun, China; ^2^ Department of Otorhinolaryngology, Head and Neck Surgery, West China Hospital, Sichuan University, Chengdu, China

**Keywords:** percutaneous nephrolithotomy, kidney stones, kidney cysts, nephrostomy, renal cyst incision, ureteral stent placement

## Abstract

Renal cysts and renal stones are common diseases in urology, but it is less common for both to coexist in a patient's kidney. In some unsophisticated urology clinics, separate elective treatments for renal cysts and renal stones are usually chosen, reducing the surgery risk but increasing the financial burden for patients. In this paper, we report a case of percutaneous nephrolithotomy for managing renal stones and endoscopy of renal cysts. The patient was a 61-year-old woman with back pain for more than 10 days. She was preoperatively diagnosed with left kidney stones and bilateral renal cysts. The patient underwent percutaneous nephrolithotomy with laser lithotripsy for left kidney stones and end cystectomy for left kidney cysts, with an indwelling ureteral stent and nephrostomy tube. The patient's back pain subsided postoperatively, and he was discharged on the 5th postoperative day. The nephrostomy tube was removed 1 week after surgery, and the ureteral stent was removed 1 month after surgery. The purpose of this case is to demonstrate to surgeons the advantages of percutaneous nephrolithotomy laser lithotripsy for treating renal stones combined with intrarenal cysts. Suppose a kidney stone is adjacent to a renal cyst, for example. In that case, if the stone is located adjacent to the renal pelvis or near the calyx in which the stone is located, percutaneous nephrolithotomy laser lithotripsy can treat both conditions.

## Introduction

Kidney stones and renal cysts are common diseases in urology, and their treatments are constantly being revolutionized with the advancement of minimally invasive techniques. Percutaneous nephrolithotripsy (PCNL) has been used as the gold standard for managing complex renal stones, with the advantages of a high stone removal rate, low trauma to the organism, and fast postoperative recovery of patients (1, 2). In recent years, the application of PCNL has been gradually expanded, not only for kidney stone treatment but also for managing renal cystic lesions such as renal cysts (3). The mainstream surgical method for renal cysts is mainly laparoscopic renal cyst decortication and decompression. Cases of simple renal cysts are treated with PCNL laser debridement and decompression, a new treatment that has been progressively proven safe and effective (4). However, there are fewer reported cases of percutaneous nephrolithotomy for the simultaneous treatment of renal stones and cysts on one side of the kidney, and the indications and outcomes of this procedure need to be further explored. In this article, we report a case of percutaneous nephrolithotomy for simultaneous management of renal stone and renal cyst endoscopy, document the treatment process of the procedure, and discuss the patient's postoperative follow-up results. We also review the relevant literature to provide reference experience in treating similar cases and explore the clinical value of PCNL in various renal diseases.

## Case presentation

**Figure 1:**
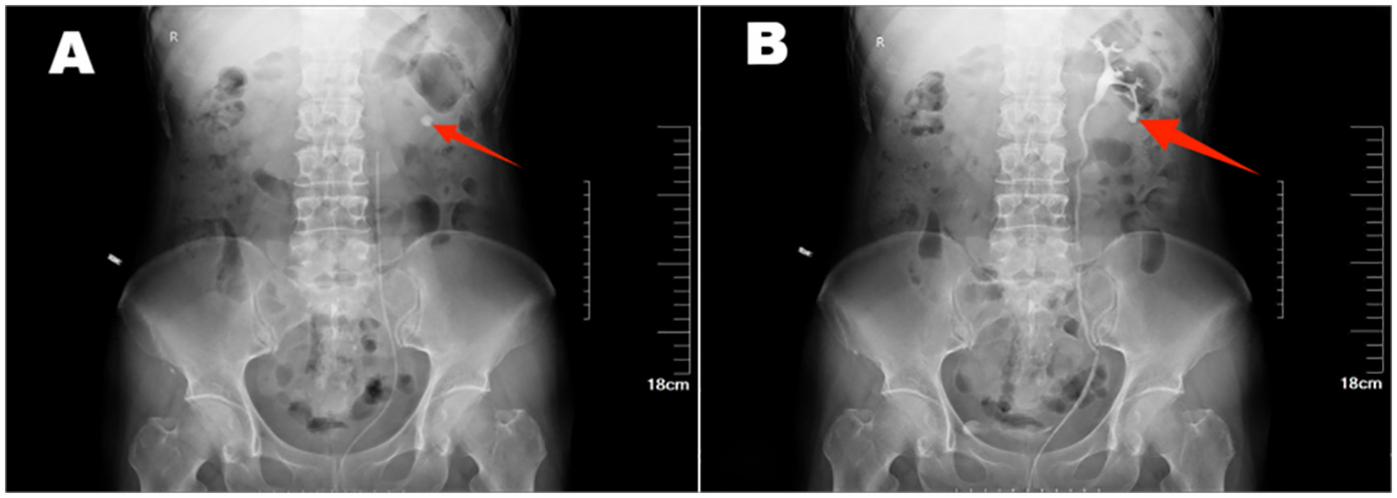
Left kidney stone is seen on unilateral retrograde urography **(A**–**B)**.

**Figure 2:**
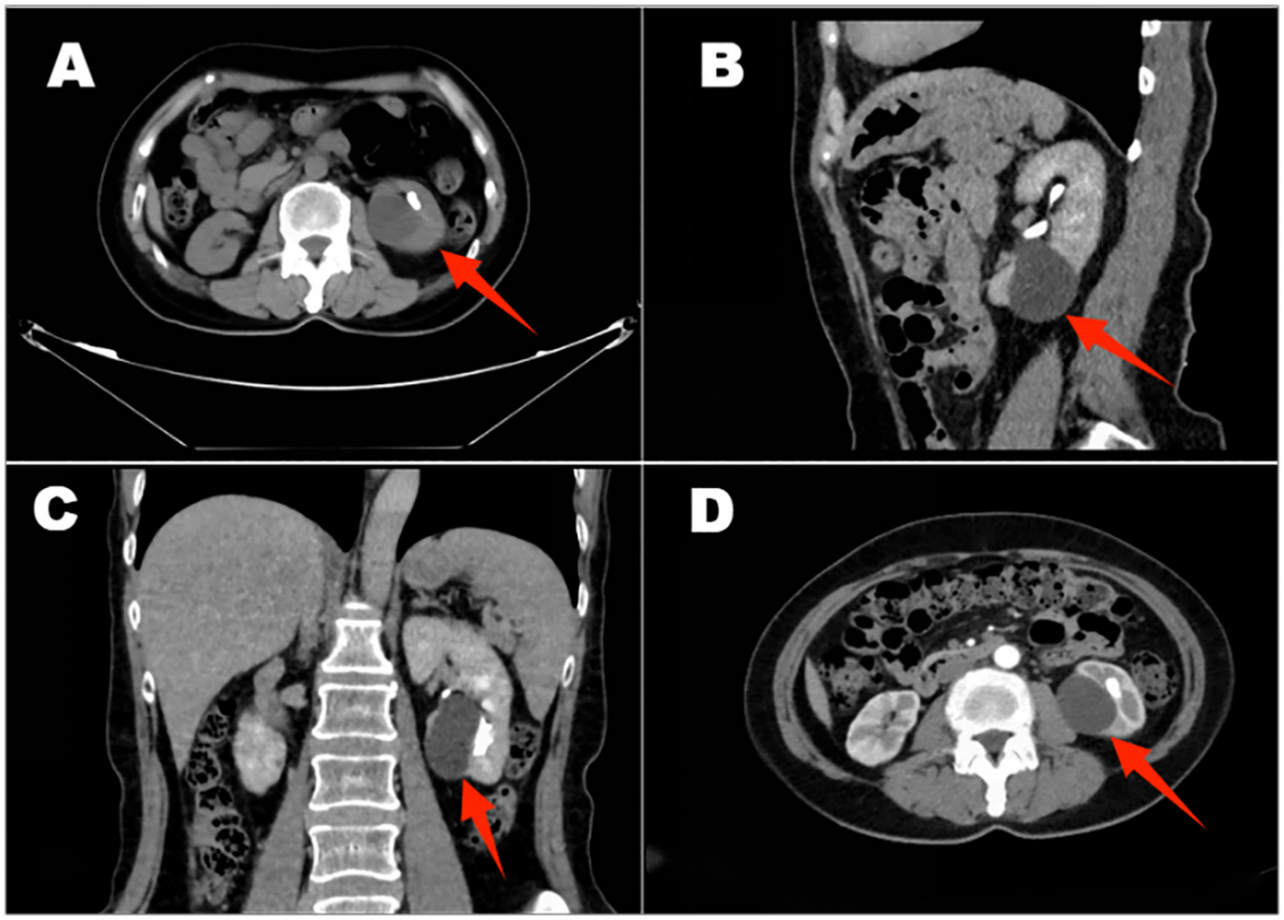
An enhanced CT scan of the kidneys shows irregular morphology, cysts in both kidneys, and more extensive lesions in the left kidney and left kidney stone **(A**–**D)**.

The patient was a 61-year-old woman who presented with posterior back pain for more than 10 days with no apparent cause. Her past medical history was normal. No similar patients have been seen in the patient's family. Physical examination on admission: bilateral lumbar curves were present symmetrically, with no tenderness at the costochondral and Costo-lumbar points and negative tenderness to percussion in both renal regions. There was no tenderness in the bilateral ureteral tracts, and the suprapubic bladder region was slightly elevated without tenderness. External genital development was normal; pubic hair was female distribution. T:36℃, P:76 beats/min, R:18 beats/min, BP:131/80 mHg, height:164 cm, weight: 59 kg.Unilateral retrograde urography: The bilateral renal contour was not clear. Nodular calcified shadows were seen in the left renal region, and no positive stone shadows were seen in the right renal region, bilateral ureteric camp alignment area, and bladder region. Retrograde urography showed that the left renal pelvis, renal calyces, and ureteric camps were well-shaped. There were no filling defects (Figure 1). Enhanced CT scanning of the kidneys: the morphology of both kidneys was irregular, and a round liquid density shadow was seen in the parenchyma of both kidneys, the size of which was about 0.5–3.7 cm, with no enhancement, and punctate calcified shadows were seen in the larger foci of the left kidney, and nodular calcified shadows in the left calyx of the left kidney, which was about 1.3 cm in size, and no dilatation of the renal monomer was seen on the two sides (Figure 2). LDL cholesterol: 3.21 mmol/L, parathyroid hormone: 77.90 pg/ml, small round epithelial cells were seen in the urine sediment: 2.7/ul, crystallography: 147.6/ul, and all other test results were within the normal range. The patient's preoperative diagnosis was left kidney stones and bilateral renal cysts.

**Figure 3:**
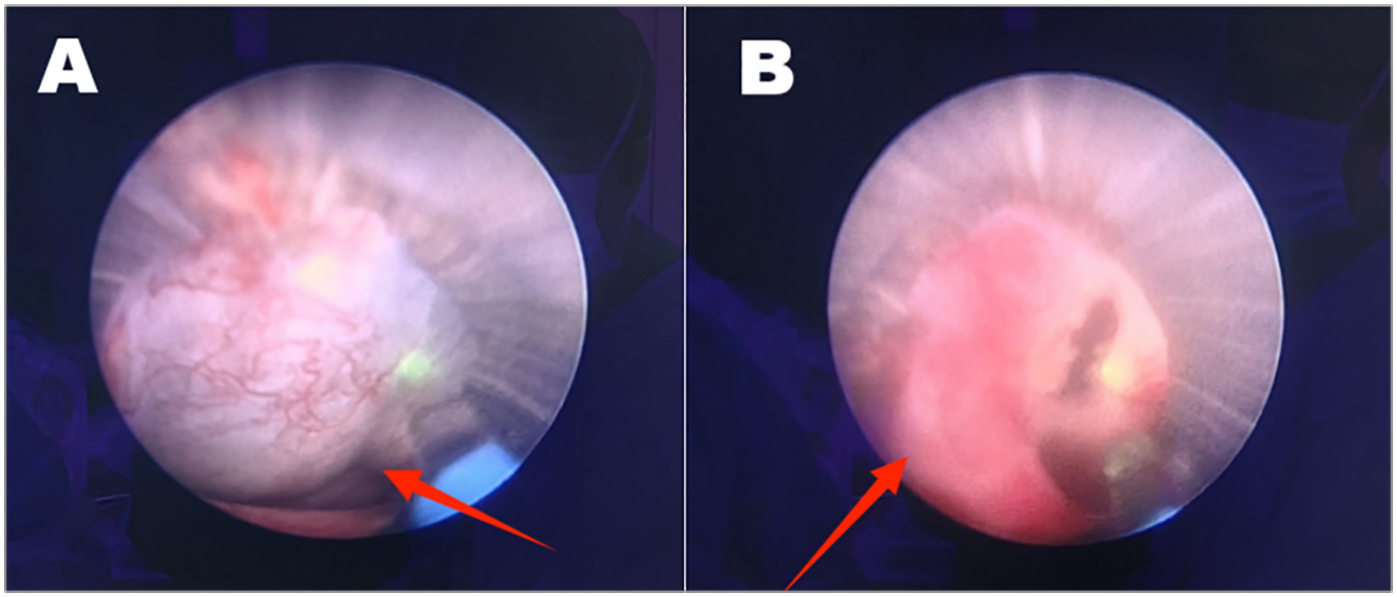
A cyst was seen next to the left lower calyx of the kidney **(A)**; the cyst’s wall was incised with a laser, it was smooth inside, and no stones or tumors were seen **(B)**.

**Figure 4:**
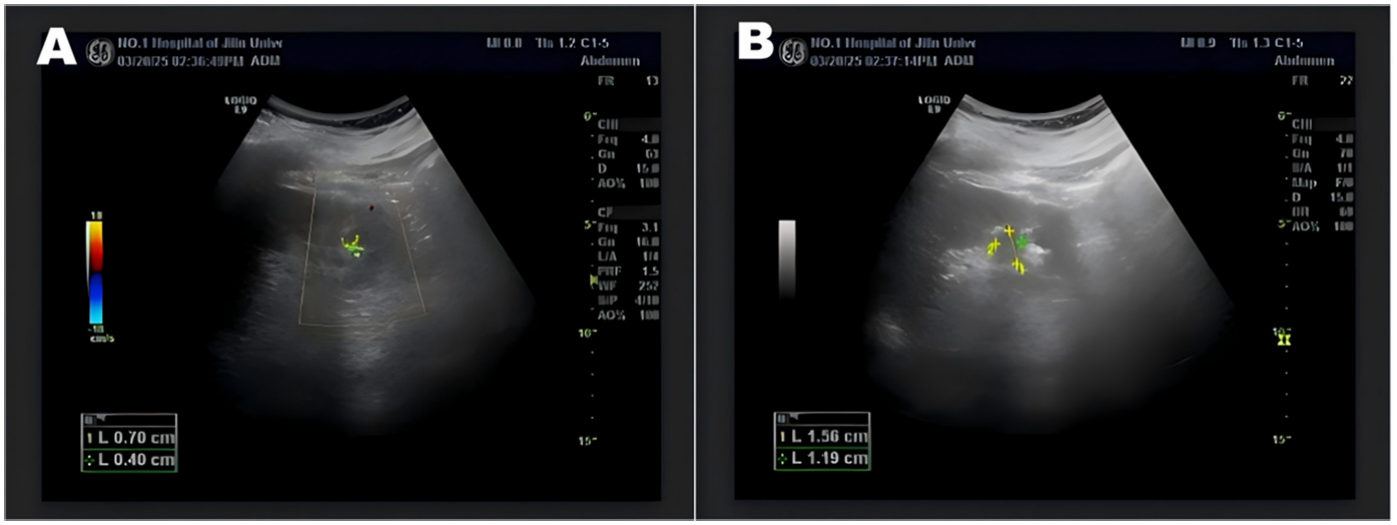
Urologic color ultrasound showed 7 mm × 4 mm left kidney stone **(A)**, anechoic left renal sinus, and tubular strong echoes in the bladder cavity **(B)**.

The patient was anesthetized in the lithotomy position and underwent cystoscopy, which showed normal bladder mucosa and no tumors or stones. A left ureteral catheter was placed under cystoscopy, and a 26 cm deep catheter was placed to retain the F16 balloon catheter and fix the ureteral catheter. Then, the patient was changed to a prone position, and the lumbar back was sterilized. In conjunction with imaging localization, the left kidney was examined by ultrasonography, which revealed strong echoes with acoustic shadows in the collecting system of the left infrarenal calyx. Saline was injected through the ureteral catheter to confirm the puncture point. The puncture needle was entered into the left lower calyx, a guidewire was placed, and the puncture channel was dilated to F16, leaving the outer sheath of the dilator in place. A percutaneous nephoscopy was performed, and a 1.5 cm yellow-brown stone was found in the left lower calyx. Holmium laser with 60 W power was used for lithotripsy, and stone fragments were drained through the percutaneous renal channel. During lithotripsy, a cyst was found adjacent to the left lower renal calyx, and the cyst's wall was incised with the holmium laser. The inner wall of the cyst was smooth, and no stones or tumors were seen (Figure 3). An F7 double-j tube was placed into the left ureter through the PCNL channel along the guidewire. The percutaneous nephoscope was withdrawn from the body, and the F16 nephrostomy tube was placed through the perforation channel at a depth of 20 cm. The F16 fistula and the perforation opening were secured using silk sutures. The patient recovered well and was discharged on the 5th postoperative day. The patient had the left nephrostomy tube removed under outpatient care 1 week after surgery. The ureteral stent was removed 1 month after surgery. Before removing the ureteral stent, the patient's urological ultrasound showed that the size of the left kidney was 99 mm × 51 mm, and the size of the right kidney was 100 mm × 50 mm. Both kidneys were normal in size and morphology, and there was no separation of the renal sinus of both kidneys, nor was there any dilation of the ureter. The left renal sinus showed an echogenic area of 15 mm × 12 mm. A strong echogenic mass with acoustic shadows was seen in the left renal sinus, measuring 7mm × 4 mm. The bladder was half-filled, and tubular echoes were seen in the bladder (Figure 4). The patient's urinary ultrasound 1 month after the operation showed small stones in the left kidney and renal cysts in the right kidney. However, the patient had no postoperative discomfort, and regular follow-up was sufficient for surgical treatment if necessary.

## Discussion

Kidney stones and simple renal cysts are both common urologic conditions that are less likely to affect younger people, but both have a higher prevalence in the older population (5, 6). However, it is rare for both diseases to occur on the same side of the kidney, and the complexity of this patient's condition makes surgical treatment riskier. Some surgeons may choose to address the problem with two separate operations. In this case, the surgeon decided to perform percutaneous nephrolithotomy with laser lithotripsy and renal cyst incision to treat the renal stone and renal cyst because of the adjacent location of the renal cyst and renal stone, which was in the lower calyces of the kidney. The etiology of kidney stones and renal cyst formation in this article's patient was considered primary hyperparathyroidism (PHPT). The parathyroid hormone can regulate the body's calcium and phosphorus metabolism, elevating blood calcium ion levels and lowering blood phosphorus levels, promoting kidney stones’ formation. The formation of stones in the kidneys may have some association with renal cysts; the two diseases can interact, but the mechanism of action is not precise; in some studies, it can be found that renal cysts can increase the risk of developing kidney stones (7, 8). In a clinically controlled study by Davut Sakız et al., the incidence of renal cysts was documented by comparing patients with primary hyperparathyroidism to healthy participants, and parathyroid hormone was found to be an independent risk factor for the development of renal cysts (9). Moreover, in this clinically controlled study, kidney stones were twice as high in PHPT patients with renal cysts as in PHPT patients without cysts (9). In this article, a patient with higher-than-normal parathyroid hormone levels was suspected of having PHPT, and the abnormal parathyroid hormone levels led to the formation of bilateral renal cysts and left kidney stones.

The mainstream surgical method for simple renal cysts is laparoscopic incision and drainage of renal cysts because this procedure is less traumatic to the body, is effective in treating renal cysts, and has a low recurrence rate after surgery (10, 11). PCNL combined with laser is also gradually used to treat renal cysts; no significant difference in therapeutic efficacy is seen between the two (12). Yang Wenzeng et al. included 32 patients with simple renal cysts treated with PCNL laser for renal cysts, all of whom experienced no postoperative adverse events (13). This study confirms the feasibility of PCNL laser treatment in patients with simple renal cysts (13). In another single-center retrospective study comparing the therapeutic efficacy and patient outcomes of laparoscopic and percutaneous nephrolithotomy laser decortication for the treatment of single renal cysts, percutaneous nephrolithotomy treatment of single renal cysts was found to be safe and effective, with lower cost-effectiveness and a lower rate of postoperative complications than laparoscopic surgery (12).

The indications for concomitant renal cyst endarterectomy during PCNL mainly depend on the patient's clinical symptoms and anatomical features. Combined surgical treatment may be considered when patients have both renal stones requiring PCNL management and renal cysts causing compression symptoms such as low back pain, hypertension, or urinary tract obstruction (14–16). If the cyst is large or located near the puncture channel, it may interfere with the PCNL operation and needs to be managed concurrently to optimize the surgical path (17, 18). Because renal cysts are located close to or within the renal pelvis, they may compress the collecting system and interfere with the surgical field of view, thereby increasing the risk of intraoperative injury to neighboring organs or urine leakage during PCNL (15, 19). When a renal cyst communicates with the renal pelvis, a percutaneous nephrolithotomy that creates a new pathway between the cyst and the renal pelvis, can effectively drain the cyst and prevent recurrence (15). It is important to ensure that the cyst is located near the PCNL puncture channel or can be managed through the same channel to avoid additional trauma (14, 20, 21). In addition, in patients with high parathyroid hormone levels, advanced age, or a history of previous stone disease, cysts and stones may be causative of each other and need to be managed at the same time (9, 22).

Placement of a ureteral stent and nephrostomy tube is recommended for adequate drainage and inspection of the cyst wall during PCNL laser management of renal cysts to avoid remaining stones and to exclude neoplastic lesions. PCNL treats both kidney stones and kidney cysts simultaneously, with a shorter postoperative recovery time, which can reduce the financial burden of patients. However, there are inevitably some surgical risks, which may lead to heavy bleeding or serious injury of kidney function due to the expanded scope of surgical operation. The simultaneous operation to relieve renal stone obstruction and renal cyst incision tends to increase the risk of urinary tract infection, so the principle of asepsis should be strictly adhered to during the operation (23). Before PCNL, CT examination and three-dimensional reconstruction of the kidneys need to be perfected to reduce the risk of the procedure and achieve a higher stone clearance rate (24, 25). Three-dimensional reconstruction can detect the anatomical relationship of renal cysts and stones to the fundamental structures within the kidney, which is essential for selecting surgical approaches in complex renal diseases (24, 25). If the cyst is located near the renal hilum and larger blood vessels within the kidney, two elective surgical procedures are recommended to minimize the risk of surgery. More minor and asymptomatic cysts are usually prioritized to deal with renal stones to relieve obstruction and then deal with renal cysts. However, secondary surgery prolongs the patient's hospitalization for a long time and causes the patient to be financially burdened. Moreover, at the interval of the second surgery, the renal cysts may increase in size and deteriorate.

Recent studies have shown that in technologically mature urological centers, the success rate of simultaneous management of stones and cysts is higher, and the incidence of postoperative complications is not significantly different from that of separate procedures (26). There is a paucity of relevant literature on the efficacy of PCNL for the surgical treatment of ipsilateral renal cysts and renal stones. In a clinical retrospective study, 28 patients with ipsilateral renal stones combined with renal cysts were included, all of whom were treated with percutaneous nephrolithotomy and laser incision of renal cysts (27). All patients had no serious perioperative complications, and the postoperative hemoglobin drop and hospital stay were within reasonable limits (27). The results of this study confirm that PCNL combined with renal cyst incision and drainage of renal cysts for the treatment of ipsilateral renal calculi and renal cysts is a feasible and safe method. However, the sample size of this study was small, and studies with larger sample sizes may be needed further to validate the safety and efficacy of this surgical approach.

## Conclusion

Percutaneous nephrolithotomy and holmium laser incision of renal cysts for ipsilateral renal calculi complicating renal cysts is a safe and effective minimally invasive treatment with great potential for treating complex renal diseases. However, its long-term therapeutic effects need further follow-up and observation.

## Data Availability

The original contributions presented in the study are included in the article/Supplementary Material, further inquiries can be directed to the corresponding author.
